# Enhanced Immunogenicity of Chicken H9N2 Influenza Inactivated Vaccine Through a Novel Dual-Targeting Fusion Protein Strategy

**DOI:** 10.3390/vaccines13030294

**Published:** 2025-03-10

**Authors:** Hai Xu, Bihua Deng, Erzhong Wu, Yalu Zhu, Qiurong Qi, Yaming Feng, Yu Lu

**Affiliations:** 1 Institute of Taizhou Agricultural Science, Jiangsu Academy of Agricultural Science, Taizhou 225300, China; hai_x@126.com; 2 GuoTai (Taizhou) Center of Technology Innovation for Veterinary Biologicals, Taizhou 225300, China; 3 Institute of Veterinary Immunology & Engineering, Jiangsu Academy of Agricultural Sciences, Nanjing 210014, China; 4 Animal Husbandry and Veterinary Department, Taizhou Municipal Bureau of Agriculture and Rural Affairs, Taizhou 225300, China

**Keywords:** dual targeting, avian influenza, dendritic cell, envelope virus

## Abstract

Background/Objectives: Targeted delivery of antigens to dendritic cells (DCs) is an effective strategy for enhancing vaccine efficacy. Methods: In this study, dual-targeting fusion proteins (GRFT-VHH54 and GRFT-VHH74) were constructed by fusing Griffithsin (GRFT), an algae-derived lectin with enveloped virus-binding properties, to DC-specific binding nanobodies (VHH54 and VHH74). Vaccines were formulated by combining the inactivated H9N2 avian influenza virus with these fusion proteins, and the potential of the fusion proteins to enhance vaccine-induced immunity in chickens was systematically evaluated. For parallel comparison, control groups included H9N2 avian influenza vaccines containing the inactivated virus alone, the inactivated virus with the immune enhancer CVCVA5, and a commercial H9N2 avian influenza inactivated vaccine. Results: At 4 weeks post-immunization, chickens vaccinated with the inactivated H9N2 virus combined with the GRFT-VHH74 fusion protein (1/2 H9+GRFT-VHH74) exhibited significantly enhanced humoral, mucosal, and cellular immune responses compared to those vaccinated with the inactivated H9N2 virus alone or the commercial H9N2 vaccine (*p* < 0.05). Additionally, chickens in the 1/2 H9+GRFT-VHH74 group exhibited enhanced resistance to the heterologous H9N2 subtype avian influenza virus, achieving a 90% protection rate, which was higher than that of the other groups. Conclusions: These results indicate that the GRFT-VHH74 fusion protein has significant potential for advancing the development of inactivated vaccines against the H9N2 subtype avian influenza. Furthermore, it provides valuable insights for enhancing the immunogenicity and efficacy of inactivated vaccines targeting other avian influenza subtypes.

## 1. Introduction

Avian influenza A viruses (AIVs) can be classified into low-pathogenic avian influenza (LPAI) and high-pathogenic avian influenza (HPAI) based on their pathogenicity to avian hosts [1]. The H9N2 avian influenza virus, a subtype of the LPAI virus, has led to decreased egg production and growth rates in chickens, resulting in significant economic losses [2]. Since its first isolation from turkeys in the USA in 1966, H9N2 AIV has spread extensively worldwide and has become endemic in land-based poultry [3,4]. In 1994, China reported the isolation of H9N2 AIV from chickens in Guangdong province, and since then, the H9N2 virus has been detected in multiple avian species, including chickens, waterfowl, and pigeons [5]. Although H9N2 infections do not cause obvious clinical signs or death in poultry, mortality can increase when a secondary infection with other pathogens occurs [6]. Moreover, H9N2 AIV has generated variants with novel antigenic and genetic characteristics, enabling the virus to cross species barriers and infect mammals without the need for intermediate hosts [5]. The expanded host range of the H9N2 virus significantly increases its potential for transmission to humans, highlighting the need for enhanced H9N2 vaccination efforts [7].

Vaccination remains an economic and effective strategy for preventing and controlling H9N2 AIV infections in poultry. Currently, two main types of commercial H9N2 vaccines are used: live and inactivated vaccines. However, the potential risks of viral contamination and genetic mutations associated with live vaccines limit their application [8]. Therefore, inactivated vaccines are most commonly used due to their ability to provide long-term protection. Moreover, a single injection of inactivated H9N2 vaccines can induce humoral and cellular immune responses [9]. In China, commercial H9N2 vaccines primarily consist of inactivated intact virus particles mixed with adjuvants to enhance immunogenicity. Although many adjuvants have been evaluated, the current oil-based commercial H9N2 inactivated vaccines still fail to provide satisfactory protection against antigenically variant viruses. The immune response induced by inactivated vaccines is weak and declines more rapidly compared to that of live vaccines, necessitating multiple doses to achieve an adequate immune response. Therefore, improving the efficacy of the currently available commercial H9N2 inactivated vaccines is essential for their field application of poultry.

**Figure 1:**
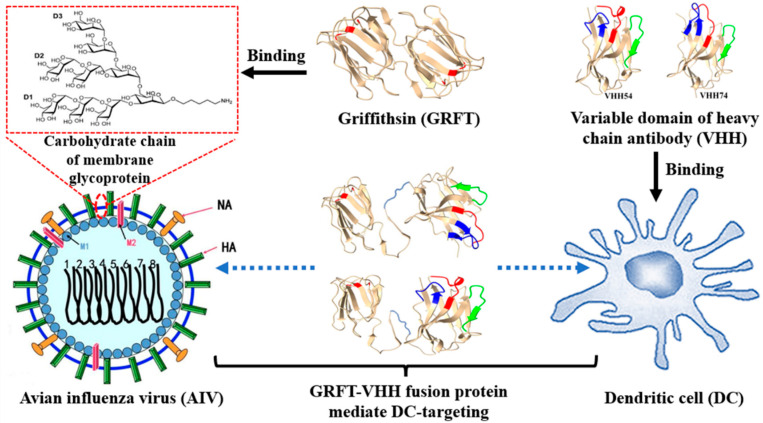
Schematic diagram of the mechanism of dual-targeting fusion protein. Griffithsin (GRFT), an algae-derived lectin, binds with carbohydrate chains of membrane glycoproteins on the membranes of various enveloped viruses. VHH54 and VHH74, derived from nanobody-specific binding with chicken dendritic cells, enable targeted interaction. The GRFT-VHH fusion protein acts as a molecular bridge, facilitating the binding between the H9N2 virus and chicken dendritic cells, thereby enhancing antigen presentation and immune response.

Dendritic cells (DCs) are highly specialized antigen-presenting cells (APCs) that can deliver diverse signals to other immune cells through intercellular interactions and soluble factors, leading to host immune responses of varying quality [10]. Delivering antigens directly to DCs in vivo by injecting antigens coupled to antibodies specific to DC surface molecules is a promising strategy for enhancing vaccine efficacy [11,12]. In our previous study, two DC-specific binding nanobodies (VHH54, VHH74) were identified from a T7 phage display nanobody library, which could potentially be used for developing DC-targeting vaccines [13]. Additionally, griffithsin (GRFT), an algae-derived lectin, has been developed as a microbicide with broad-spectrum activity through its ability to bind glycans on several enveloped viruses [14]. These findings inspired us to create a dual-targeting protein capable of simultaneously binding H9N2 AIV and dendritic cells. Through the bridging effect of this dual-targeting protein, the capture, processing, and presentation of the inactivated H9N2 virus by dendritic cells could be promoted, ultimately improving immune efficiency (Figure 1). In this study, dual-targeting proteins were constructed by linking DC-targeting nanobodies with GRFT and subsequently incubated with inactivated H9N2 virus to prepare oil emulsion vaccines. Specific pathogen-free (SPF) chickens were immunized to evaluate humoral and cellular immune responses, and the vaccine’s protective efficacy against the virus was verified.

## 2. Materials and Methods

### 2.1. Construction of Recombinant Plasmids

The genes encoding GRFT-VHH54 and GRFT-VHH74 were synthesized (GenScript, Nanjing, China) and cloned into the prokaryotic expression vector pET-32a (+) using *Nde*I and *Xho*I restriction enzyme sites. The resulting recombinant plasmids, pET-GRFT-VHH54 and pET-GRFT-VHH74, were subsequently transformed into *E. coli* BL21(DE3) competent cells. Positive transformants were selected on LB agar plates containing ampicillin. Colony PCR, restriction digestion, and DNA sequencing were performed to confirm the successful insertion of the target genes.

### 2.2. Preparation and Identification of Dual-Targeting Protein

The pET-GRFT-VHH54 and pET-GRFT-VHH74 clones were induced with 0.5 mM IPTG at an optical density (OD) of 0.5 at 600 nm and incubated at 20 °C for 18 h. After induction, cells were harvested and resuspended in a binding buffer (20 mM sodium phosphate, 500 mM NaCl, 20 mM imidazole, pH 7.4). Cells were lysed by sonication, and the lysates were cleared by centrifugation at 14,000× *g* for 30 min. The supernatants were applied to HisTrap HP His-tag protein purification columns (GE Healthcare, Chicago, IL, USA). After washing with a binding buffer containing 40 mM imidazole, the recombinant proteins were eluted with 250 mM imidazole. Protein purity and molecular weight were assessed via SDS-PAGE, and Western blot analysis was performed using HRP-conjugated Anti-6X His Tag antibody (Abcam, Cambridge, UK) (1:5000). Homology modeling of VHH54, VHH74, and GRFT was conducted using SWISS-MODEL with templates 7TGF, 6H71, and 7RKG templates, respectively [15,16]. UCSF ChimeraX 1.5 was used for structure visualization [17].

### 2.3. Quantitative and Qualitative Analysis of Dual-Targeting Protein Interaction with H9N2 Virus

The H9N2 AI virus strain (NJ01) was adjusted to a titer of 10^8^ EID_50_/0.1 mL and subjected to two-fold serial dilutions. Ten microliters of each dilution was spotted onto a nitrocellulose membrane. Phosphate-buffered saline (PBS), GRFT-VHH54, and GRFT-VHH74 proteins were included as negative and positive controls. The membrane was blocked overnight in 1% skim milk and then incubated with 2 μg/mL GRFT-VHH54 and GRFT-VHH74 proteins for 45 min. After washing with PBS that contains 0.1% Tween-20 (PBST), the membrane was incubated with HRP-conjugated Anti-6X His Tag antibody (1:5000) for 45 min, followed by color development with DAB substrate.

A 96-well ELISA plate was coated with H9N2 virus at 10^8^ EID_50_/0.1 mL and blocked overnight with 1% skim milk. GRFT-VHH54 and GRFT-VHH74 proteins, prepared at 2 μg/mL, were serially diluted two-fold, and 100 μL of each dilution was added to the wells for 45 min. After washing with PBST, 100 μL of HRP-conjugated Anti-His Tag antibody (1:10,000) was added for 45 min. Plates were washed, developed with 3,3′,5,5′-Tetramethylbenzidine (TMB) substrate, and stopped with 2 M H_2_so_4_, and absorbance was measured at 450 nm.

### 2.4. Interaction of Dual-Targeting Proteins with Chicken Bone Marrow Dendritic Cells

Chicken bone marrow-derived dendritic cells were prepared as previously described [18,19]. On day 7, dendritic cells were seeded onto cell slides and incubated at 37 °C with 5% CO_2_ until approximately 70% confluence. The cells were fixed with 4% paraformaldehyde for 15 min at room temperature, permeabilized with 0.1% Triton X-100 for 10 min, and blocked with 5% BSA for 1 h. After three washes, the cells were incubated with 200 ng/mL GRFT, GRFT-VHH54, and GRFT-VHH74 for 45 min. Unbound proteins were washed away, and an Alexa Fluor^®^ 488 Anti-6X His tag antibody (Abcam, UK; 1:5000) was added for 45 min. Phalloidin-iFluor 594 Conjugate (abs42235791, Absin, Shanghai, China; 1:1000) was applied for 1 h in the dark. Coverslips were mounted with VECTA shield mounting medium (Vector laboratories, Burlingame, CA, USA) with 4’,6-diamino-2-phenylindole (DAPI), and slides were visualized with a ZEISS confocal microscope (ZEISS LSM 880, ZEISS, Oberkochen, Germany) at the Yangzhou University’s Testing & Analysis Center.

### 2.5. H9N2 Inactivate Vaccines Preparation and Chicken Immunization

The H9N2 AI virus, with a titer of 4 × 10^8^ EID_50_/mL, was inactivated using 0.1% binary ethyleneimine (BEI). The inactivated virus was then emulsified with ISA 206 adjuvant at a 46:54 (*v*/*v*) ratio to produce a water-in-oil-in-water emulsion H9 vaccine. Additionally, the inactivated H9 virus was incubated with equal volumes of GRFT-VHH54 and GRFT-VHH74 for 1 h, with each protein at a concentration of 1 µg/mL. These mixtures were subsequently emulsified with ISA 206 adjuvant at the same 46:54 (*v*/*v*) ratio to produce the H9+GRFT-VHH54 and H9+GRFT-VHH74 vaccines. The CVCVA5 immune booster [20,21], provided by the Jiangsu Academy of Agricultural Sciences, comprised an emulsion containing poly I:C, the MDPL-D isoform, and levamisole hydrochloride. SPF White Leghorn chickens (*G. gallus domesticus*) were housed in isolation units from 1-day post-hatching. At 2 weeks of age, seven groups, each comprising fifteen chickens, were immunized subcutaneously with the prepared vaccines. The detailed composition of these vaccines and the immunization protocols followed for each group were provided in Table 1.

### 2.6. Detection of Antibodies and Cytokines

Blood samples were collected at 2 and 4 weeks post-immunization. Serum antibody titers were measured using a hemagglutination inhibition (HI) assay. Four weeks post-immunization, five chickens from each immunization group were randomly selected for euthanasia. Mucosal antibodies in tracheal mucus, bronchoalveolar lavage (BAL) fluids, and small intestine mucus were assessed using the HI assay, as described previously. Briefly, BAL fluids were collected to obtain tracheal mucus by instilling 10 mL of phosphate-buffered saline (PBS, pH 7.2) into the bronchus and lungs using a 20 mL syringe, followed by aspiration. This procedure was repeated 10 times, and 1 mL of BAL fluid was retained for antibody titer measurement. Tracheal mucus was scraped from tracheal sections and rinsed with 0.5 mL PBS. Intestinal mucus was collected from a 10 cm segment of the small intestine after the contents were cleared, followed by scraping and transferring the mucus into 2 mL ice-cold PBS. The mucus samples were centrifuged at 10,000× *g* for 15 min at 4 °C to remove particulates. The resulting supernatant, containing crude intestinal mucus, was used for HI testing. In addition, the levels of T-helper type 1 cytokine IFN-γ and Th2-type cytokine IL-4 in serum were measured at 4 weeks post-immunization using commercially available ELISA kits (AndyGene, Beijing, China) according to the manufacturer’s instructions.

### 2.7. Lymphocyte Proliferation Assay

Lymphocyte proliferation was assessed using the CCK-8 assay. Peripheral blood mononuclear cells (PBMCs) were isolated by density gradient centrifugation with a lymphocyte isolation kit (TBD, Tianjin, China) and washed twice with fresh RPMI 1640 medium (Gibco, Waltham, MA, USA). The cells were resuspended at 5 × 10^5^ cells/mL in RPMI 1640 medium supplemented with 10% fetal bovine serum (FBS), 100 units/mL penicillin, and 100 μg/mL streptomycin. The cells were cultured in 96-well plates and stimulated for 72 h at 37 °C in a 5% CO_2_ incubator. Concanavalin A (Con A, 5 μg/mL, Sigma, St. Louis, MO, USA) was used as a positive control, while purified inactivated H9N2 virus (5 μg/mL) served as the specific antigen. An untreated culture served as the negative control. Following stimulation, 10 μL of CCK-8 solution was added to each well, and the cells were incubated for an additional 4 h. The optical density (OD) at 450 nm was measured using a microplate reader.

### 2.8. Virus Challenge of Immunized Chickens

At 28 days post-vaccination (dpv), ten chickens in each group were intranasally challenged with 0.1 mL of a heterologous H9N2 subtype AI virus (HN03) at a dose of 10^7^ EID_50_. The chickens were observed for clinical signs over a 14-day monitoring period. At the end of the observation period, surviving birds were humanely euthanized for gross lesion examination. Oropharyngeal and cloacal swabs were collected at 3, 5, and 7 days post-challenge (dpc) or immediately upon death during the observation period. Virus isolation from swab samples was performed as described in a previously published protocol [22].

### 2.9. Statistical Analysis

Data from antibody, cytokine, and lymphocyte proliferation assays were analyzed using SPSS software (version 28). Statistical comparisons between groups were performed using a one-way analysis of variance (ANOVA) followed by Tukey’s post hoc test. Differences between groups were denoted by letters. Results with a *p*-value of less than 0.05 were considered statistically significant. Data were expressed as the mean ± standard deviation (S.D.) for each group.

## 3. Results

### 3.1. The Prokaryotic Expression Vector Successfully Induced the Expression of the Dual-Targeting Fusion Protein

**Figure 2:**
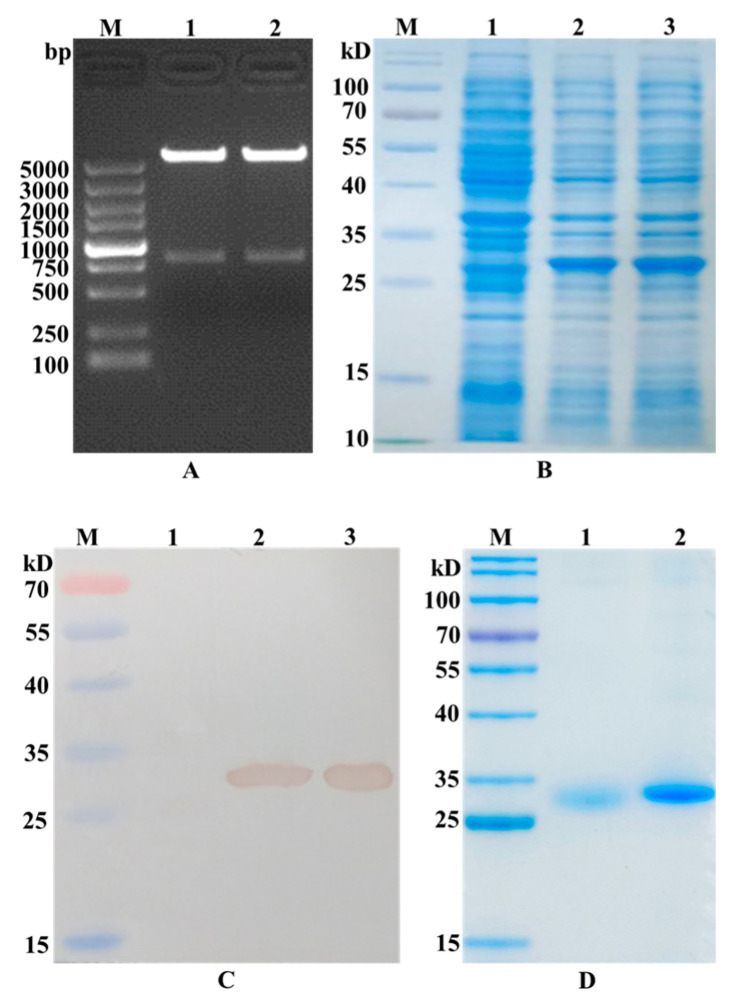
Identification of recombinant plasmid construction and dual-targeting protein expression. (**A**) Double digestion analysis of the recombinant plasmid pET-GRFT-VHH54 and pET-GRFT-VHH74. Line M, DL5000 marker; Line 1, pET-GRFT-VHH54 double digestion with *Nhe*I and *Xho*I; Line 2, pET-GRFT-VHH74 double digestion with *Nhe*I and *Xho*I. (**B**) Analysis of fusion protein expression by SDS-PAGE. Line M, pre-stained protein molecular weight marker (10 to 180 kDa, Fermentas); Line 1, uninduced *E. coli* BL21 host; Line 2, recombinant bacteria expressed GRFT-VHH54 fusion protein; Line 3, recombinant bacteria expressed GRFT-VHH74 fusion protein. (**C**) Identification of fusion protein by Western blot. Line M, pre-stained protein molecular weight marker (10 to 180 kDa, Fermentas); Line 1, uninduced *E. coli* BL21 host; Line 2, fusion protein GRFT-VHH54; Line 3, fusion protein GRFT-VHH74. (**D**) Analysis of purified fusion protein by SDS-PAGE. Line M, pre-stained protein molecular weight marker (10 to 180 kDa, Fermentas); Line 1, fusion protein GRFT-VHH54; Line 2, fusion protein GRFT-VHH74.

The GRFT-VHH54 and GRFT-VHH74 genes were successfully inserted into the multiple cloning site (MCS) of the pET-32a (+) vector, as confirmed by the presence of a 790 bp fragment following double digestion with *Nde* I and *Xho* I (Figure 2A). After IPTG induction, significant expression of the GRFT-VHH54 and GRFT-VHH74 fusion proteins was observed, as evidenced by a prominent band at the expected 27 kDa on SDS-PAGE gels (Figure 2B). The fusion proteins were subsequently purified using HisTrap affinity columns, with successful purification confirmed by the appearance of a single strong band on SDS-PAGE (Figure 2C). Western blot analysis further validated the identity of the purified proteins, displaying a single band at the expected molecular weight using an anti-His antibody (Figure 2D). These findings demonstrate that the constructed recombinant expression vectors effectively induced the expression of fusion proteins.

### 3.2. The Three-Dimensional Structure of Proteins Revealed the Potential of Binding Ability

**Figure 3:**
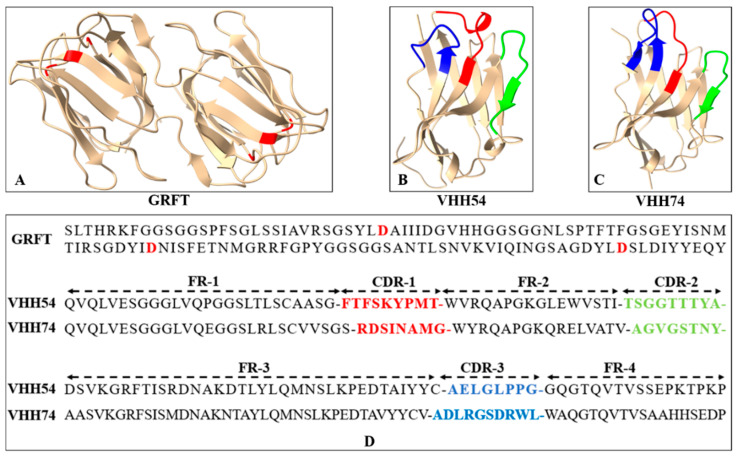
Sequence and three-dimensional structure of Griffithsin (GRFT) and nanobodies. (**A**) Ribbon drawing showing the three-dimensional structure of GRFT. Two GRFT monomers are presented in yellow. The aspartic acid residues critical for mannose-binding within the three carbohydrate-binding domains are highlighted in red. (**B**,**C**) Three-dimensional structure of VHH54 and VHH74. The yellow ribbon represents framework regions in each domain. The three complementarity determining regions (CDR1-3) are colored red, green, and blue. (**D**) Amino acid sequences of GRFT, VHH54, and VHH74. The aspartic acid residues for mannose-binding in GRFT are highlighted in red. The CDR1-3 of VHHs are colored red, green, and blue.

The three-dimensional structures of GRFT, VHH54, and VHH74 proteins were modeled using SWISS-MODEL, demonstrating well-folded conformations. The GRFT model displayed characteristic dimer formation (Figure 3A) with three repeats of antiparallel four-stranded β-sheets forming a triangular prism shape. The VHH54 and VHH74 nanobodies showed exposed loop structures in their complementarity-determining regions (CDRs) (Figure 3B,C). These structure characteristics of VHHs suggest an optimal configuration for interacting with dendritic cell surface receptors, potentially enhancing targeting efficiency. The amino acid sequences of GRFT, VHH54, and VHH74, with key binding residues and CDRs highlighted, are presented in Figure 3D. The predicted models displayed characteristic secondary structure elements consistent with the known architectures of Griffithsin and VHH domains, as visualized in the generated 3D structures.

### 3.3. Dual-Targeting Fusion Protein Efficiently Bounded to H9N2 Avian Influenza Virus

**Figure 4:**
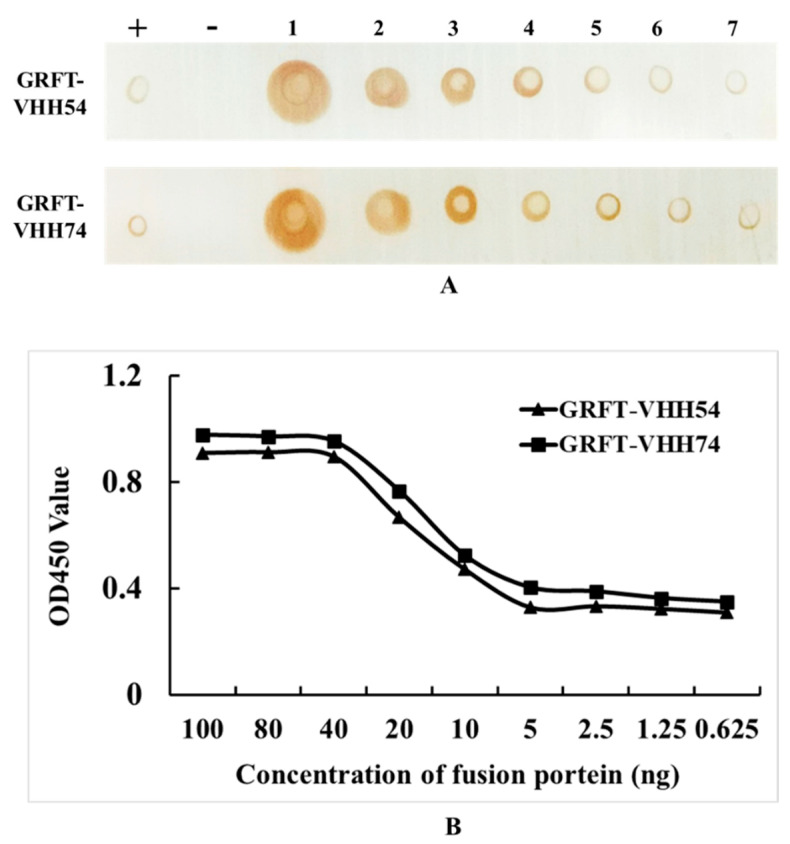
Quantitative and qualitative analysis of dual-targeting proteins interacting with H9N2 virus. (**A**) Quantitative analysis: (+) indicates GRFT-VHH54 and GRFT-VHH74 positive control; (−) indicates PBS negative control; (1–7) represents two-fold serial dilutions of H9N2 virus, starting from a titer of 10^8^ EID_50_/0.1 mL, spotted onto a nitrocellulose membrane. (**B**) Qualitative analysis: ELISA plate was coated with H9N2 virus (10^8^ EID_50_/0.1 mL) and interacted with serially diluted GRFT-VHH54 and GRFT-VHH74 fusion proteins.

In the Dot blot assay, serial dilutions of GRFT-VHH54 and GRFT-VHH74 exhibited a dose-dependent signal intensity (Figure 4A). Higher concentrations produced robust signals (Dot 1), whereas lower concentrations showed progressively weaker signals (Dot 7). Positive controls displayed distinct spots, confirming the specificity of the assay, while negative controls exhibited no detectable signal, validating its accuracy. These results indicate a strong binding affinity of the dual-targeting fusion proteins to H9N2 viruses. Furthermore, quantitative ELISA demonstrated a dose-dependent increase in OD450nm values as varying concentrations of the fusion proteins interacted with H9 virus titers (Figure 4B). At fusion protein concentrations exceeding 40 ng, complete binding to 10^8^ EID_50_ H9N2 virus was observed, with OD values plateauing beyond this concentration. In contrast, concentrations below 5 ng resulted in only background binding, with no further reduction in OD values upon additional dilution. This quantitative binding relationship provides essential insights for optimizing vaccine formulation.

### 3.4. Dual-Targeting Fusion Protein Efficiently Bounded to Chicken Bone Marrow-Derived Dendritic Cells

**Figure 5:**
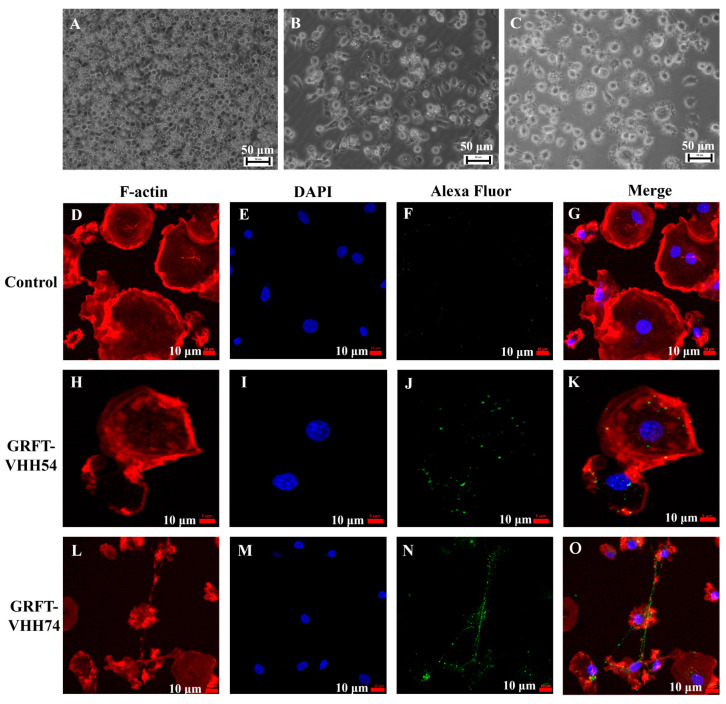
Interaction between dual-targeting proteins and chicken dendritic cells detected by laser confocal microscopy. Morphology of chicken bone marrow dendritic cells (DCs) on days 2, 4, and 6 (**A**–**C**). Binding of GRFT-VHH54 and GRFT-VHH74 fusion proteins to DCs was visualized with Alexa Fluor^®^ 488 anti-6X His tag antibody (green) (**F**,**J**,**N**). Actin filaments were stained with Phalloidin-iFluor 594 (red) (**D**,**H**,**L**) and nuclei with DAPI (blue) (**E**,**I**,**M**). Merged images are shown in (**G**,**K**,**O**).

Chicken bone marrow-derived dendritic cells were induced and utilized to evaluate the binding ability of the dual-targeting fusion proteins. The morphological and phenotypic changes observed during the in vitro differentiation of dendritic cells are depicted in Figure 5. At the early stage, dendritic cells appeared as small, round, or oval cells (Figure 5A). As differentiation progressed, the cells began to elongate, developing pseudopodia and dendrite-like processes (Figure 5B). In the final stage, dendritic cells exhibited more complex morphologies and irregular cell shapes (Figure 5C). The binding of dual-targeting fusion proteins to dendritic cells was assessed using fluorescent microscopy. No green fluorescent signal was detected in the control group (Figure 5F). In contrast, GRFT-VHH54 and GRFT-VHH74 efficiently adsorbed to the dendritic cell surface, as evidenced by the strong green fluorescence signals covering the dendritic cell surface (Figure 5J,N).

### 3.5. Immunization of Chickens with Dual-Targeting Protein Chimeric H9 Vaccines Induced Enhanced Antibody Responses

**Figure 6:**
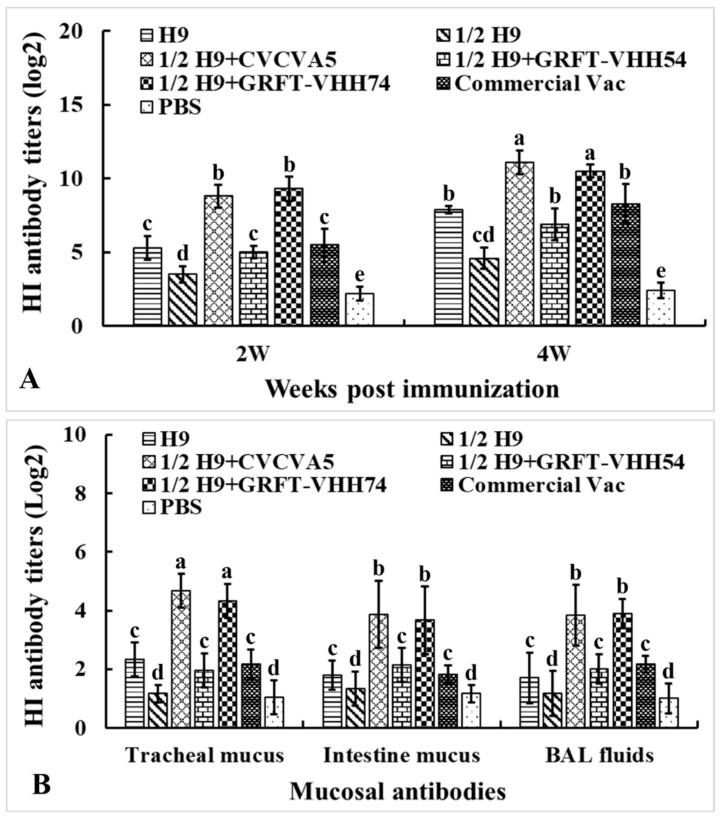
Hemagglutination inhibition (HI) antibody titers against H9 viral antigen. Specific pathogen-free (SPF) White Leghorn chickens (*n* = 15) were immunized at 2 weeks of age, and sera were collected at 2 and 4 weeks post-immunization. Mucosal samples (*n* = 5) were collected at 4 weeks post-immunization. (**A**) HI antibody titers in serum at 2 and 4 weeks post-immunization. (**B**) HI antibody titers in intestinal wash, tracheal wash, and bronchoalveolar lavage (BAL) fluids. Bars with different letters indicate significant differences (*p* < 0.05). Values represent mean ± S.D.

To evaluate the humoral and mucosal immune responses elicited by different immune enhancers formulated with H9 vaccines, sera samples were collected at weeks 2 and 4, and mucosal samples at week 4 post-immunization. The samples were analyzed using HI testing (Figure 6A). At both weeks 2 and 4, HI antibody titers against the H9N2 virus in the 1/2H9+CVCVA5 and 1/2H9+GRFT-VHH74 groups were significantly higher compared to the H9, 1/2H9, 1/2H9+GRFT-VHH54, Commercial Vac, and PBS control groups (*p* < 0.05). However, no significant differences in HI antibody titers were observed among the 1/2H9+GRFT-VHH54, H9, and Commercial Vac groups. Interestingly, chickens vaccinated with 1/2H9-GRFT-VHH54 still showed significantly higher HI antibody titers than those in the 1/2H9-only group (*p* < 0.05). At 4 weeks post-immunization, mucosal samples from the trachea, small intestine, and BAL fluids were collected for HI antibody measurement (Figure 6B). Notably, chickens administered the H9 vaccine with either CVCVA5 or GRFT-VHH74 as immune enhancers exhibited significantly higher mucosal antibody levels compared to other groups. These findings, consistent across both serum and mucosal antibody data, indicated that GRFT-VHH74 effectively enhances the humoral immune response to the H9 vaccine, demonstrating efficacy comparable to that of CVCVA5.

### 3.6. The H9 Vaccine Supplemented with Dual-Targeting Protein Enhanced Cellular Immune Responses

**Figure 7:**
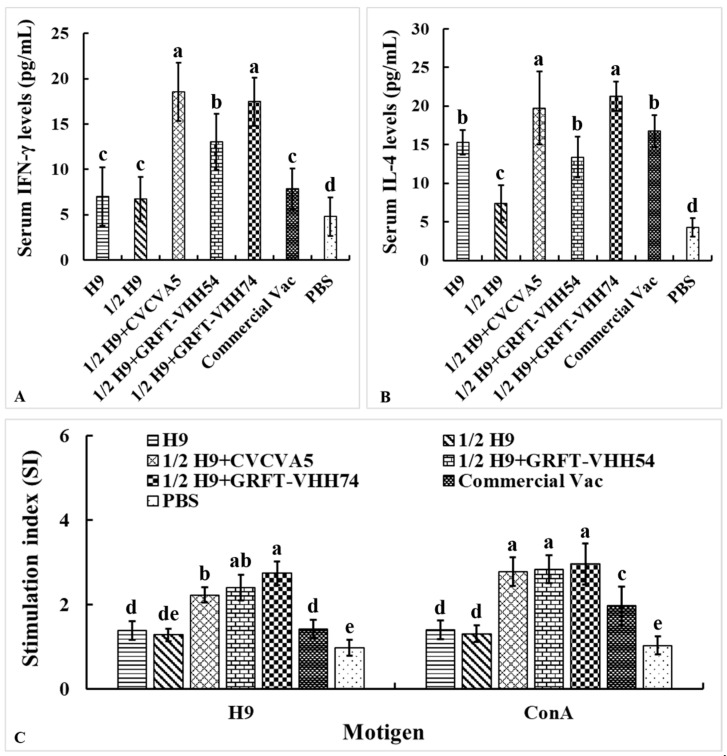
Evaluation of serum cytokine levels and lymphocyte stimulation index. The Th1 (**A**) and Th2 (**B**) cell-associated cytokines released into the blood at 4 weeks post-immunization were evaluated using commercial chicken cytokine detection kits (AndyGene, China). Peripheral blood lymphocytes (PBMCs) were prepared at 4 weeks post-immunization and cultured with H9N2 virus (5 μg/mL) and Con A (5 μg/mL). Lymphocyte proliferation was measured by CCK-8 reagent as described in the text and shown as stimulation index. (**A**) Gamma interferon (IFN-γ). (**B**) Interleukin-4 (IL-4). (**C**) PBMCs stimulation index. Values represent mean ± S.D. (*n* = 15). Bars with different letters indicate significant differences (*p* < 0.05).

The levels of Th1-type cytokine IFN-γ and Th2-type cytokine IL-4 in serum were measured to evaluate the cellular immune responses induced by the H9 vaccines. At 4 weeks post-vaccination, chickens immunized with H9 vaccine supplemented with either CVCVA5 or GRFT-VHH74 exhibited significantly higher levels of IFN-γ and IL-4 compared to those immunized with H9 vaccine alone or supplemented with GRFT-VHH54 (Figure 7A,B). These results demonstrated the potential of GRFT-VHH74 to enhance both Th1 and Th2 immune responses. Additionally, lymphocyte proliferation assays revealed significantly higher stimulation indices (SIs) in chickens immunized with 1/2 H9+GRFT-VHH74 compared to 1/2 H9, 1/2 H9+CVCVA5, Commercial Vac, and PBS controls when lymphocytes were stimulated with H9 virus antigen (*p* < 0.05) (Figure 7C). However, no significant differences in SI were observed among the 1/2 H9+CVCVA5, 1/2 H9+GRFT-VHH54, and 1/2 H9+GRFT-VHH74 groups when lymphocytes were stimulated with ConA. This finding suggested that ConA induced non-specific T-cell activation, while H9-specific proliferation highlighted the ability of the vaccine supplemented with GRFT-VHH74 to elicit a targeted immune response.

### 3.7. Enhanced Protection Against Heterologous H9 Virus Challenge with Dual-Targeting Protein Supplemented H9 Vaccine

The challenge was performed at 4 weeks post-vaccination using a heterologous H9N2 subtype AI virus (HN03) at a dose of 10^7^ EID_50_. No mortalities occurred in the immunized groups, whereas one chicken in the PBS control group succumbed on day 8 post-challenge. Mild clinical signs, including transient depression, were observed in chickens from the 1/2H9+CVCVA5 and 1/2H9+GRFT-VHH74 groups, lasting only three days. In contrast, chickens in other groups exhibited more severe and prolonged symptoms, particularly in the 1/2H9 group. Virus isolation from oropharyngeal and cloacal swabs collected on days 3, 5, and 7 post-challenge (dpc) revealed significantly reduced viral shedding in the 1/2H9+CVCVA5 and 1/2H9+GRFT-VHH74 groups compared to the 1/2 H9 group. Specifically, by day 5, 7 out of 10 chickens in the 1/2 H9 group were still shedding the virus, whereas no virus shedding was detected in chickens from the 1/2H9+CVCVA5 and 1/2H9+GRFT-VHH74 groups by day 7. These findings highlight the enhanced protective efficacy of the H9 vaccine formulations supplemented with CVCVA5 or GRFT-VHH74 (Table 2).

## 4. Discussion

H9N2 AIV is classified as a low-pathogenic virus; however, it exerts significant negative impacts on poultry health by impairing the immune system, increasing vulnerability to secondary infections, and causing considerable economic losses in the poultry industry [23]. Since the first H9 inactivated vaccine was approved in 1998, a variety of monovalent and combination vaccines have been deployed in China, encompassing up to 25 different vaccine strains used in licensed H9 vaccine products [24]. Similar vaccination strategies are employed in countries, such as Mexico, Egypt, and Pakistan, where adjuvanted inactivated whole virus vaccines remain the predominant approach [25]. While these vaccines have successfully mitigated clinical symptoms and reduced the transmission of H9N2, challenges persist in achieving optimal efficacy [5,26]. Notably, current vaccines often exhibit limited capacity to elicit robust cellular and mucosal immune responses, which are critical for comprehensive protection against viral infections. Consequently, there is a pressing demand for improved vaccine formulations capable of inducing balanced humoral, cellular, and mucosal immunity to enhance protective outcomes.

DCs are highly specialized antigen-presenting cells that play a crucial role in maintaining both innate and adaptive immune responses. Previous studies have demonstrated that antibody-mediated targeting of proteins to DC surface receptors significantly enhances antigen immunogenicity in vivo, leading to stronger immune activation and responses [27]. Among these receptors, DEC-205, abundantly expressed on DCs, was the first to be leveraged for targeted antigen delivery. Notably, a single dose of an anti-DEC-205 antibody conjugated with hemagglutinin induced a robust immune response in chickens as early as 14 days post-priming [28]. In this study, we employed a dual-targeting strategy combining viral and dendritic cell-binding domains to enhance vaccine immunogenicity. Nanobodies, selected via bio-panning from a T7 phage-displayed nanobody library, were specifically designed to bind chicken bone marrow-derived DCs [13]. Simultaneously, GRFT was utilized to target viral envelope glycoproteins by binding to the terminal mannose residues of high mannose oligosaccharides on the virus surface [29]. This dual-targeting approach aimed to synergistically improve antigen delivery to DCs and enhance immune responses. The expression and purification of GRFT-VHH fusion proteins were successfully achieved, and in vitro assays confirmed their strong binding affinity to both H9 AIV and chicken DCs. These findings highlight the potential of this strategy to improve antigen presentation and immune activation. Moreover, this dual-targeting approach offered a promising framework for the development of next-generation poultry vaccines, with potential applications extending to other species and vaccine platforms.

Inactivated AIV vaccines are well documented for their ability to elicit robust serum antibody responses; however, they are markedly less effective at inducing mucosal and cell-mediated immune responses [30]. Additionally, these vaccines fail to activate pattern-recognition receptors (PRRs) on infected cells [31], a crucial step for amplifying proinflammatory cytokines and type I interferon production, which derives the local cellular innate immune response [32]. To address these limitations, we developed and evaluated a novel H9 vaccine formulation supplemented with dual-targeting fusion proteins (GRFT-VHH54 and GRFT-VHH74). Immunization with dual-targeting protein-supplemented H9 vaccine resulted in significantly higher serum HI antibody titers and enhanced mucosal immune responses compared to the groups receiving the H9 vaccine alone or other formulations. Notably, the cellular immune response was also improved, as evidenced by elevated levels of IFN-γ and IL-4 cytokines and enhanced lymphocyte proliferation in the GRFT-VHH74 supplemented group. These findings suggest that GRFT-VHH74 enhances both Th1 and Th2 responses, providing balanced and targeted immune activation—a critical feature for achieving protective efficacy against H9N2 AIV. This dual-targeting strategy represents a promising approach to overcome the inherent limitations of conventional inactivated H9 vaccines, offering a more comprehensive and effective immune defense.

A two-dose regimen of the inactivated H9 vaccine is typically required to elicit a robust immune response in broiler chickens [33]. However, due to the shorter lifespan of broilers compared to laying hens, achieving a rapid and efficient immune response post-vaccination is particularly critical [34]. Moreover, the antibodies induced by conventional inactivated H9 vaccines often fail to provide full protection against homologous virus infections under field conditions [35,36]. To evaluate the enhanced protective efficacy of the dual-targeting fusion protein-supplemented H9 vaccine, chickens were challenged with a heterologous H9N2 virus (HN03). Immunized chickens receiving the dual-targeting formulations exhibited significantly reduced viral shedding and milder clinical symptoms compared to control groups. Notably, chickens immunized with the GRFT-VHH74 supplemented vaccine showed nearly complete protection, with minimal viral shedding and negligible clinical symptoms. However, neither anatomical examination of the respiratory system nor histopathological analysis of visceral organs was performed on the challenged chickens. Still, the results from virus isolation were enough to highlight the potential of GRFT-VHH74 to significantly enhance the protective efficacy of H9 vaccines, offering a more effective solution for broiler flocks with shorter production cycles and heightened susceptibility to infection.

Compared to previous studies, our dual-targeting approach offers several distinct advantages. Conventional inactivated H9 vaccines often rely on higher doses or multiple administrations to achieve optimal immune responses [37,38]. The 1/2 H9+GRFT-VHH74 group, demonstrated superior protective efficacy with single-dose immunization despite containing merely 10% antigen content relative to commercial H9 vaccine. This group also exhibited accelerated immune activation, achieving significantly elevated antibody titers by week 2 post-vaccination compared to both the monovalent H9 group and commercial vaccine group. Crucially, our approach significantly enhances both innate and adaptive immunity, with a notable improvement in mucosal immunity—a critical defense mechanism against respiratory pathogens like H9N2. Targeting dendritic cells has been widely recognized as a strategy to improve vaccine efficacy [39,40]. Our results build on this concept by showcasing the exceptional targeting efficiency of GRFT-VHH74, which not only facilitates precise antigen delivery to DCs but also amplifies downstream immune responses.

## 5. Conclusions

In summary, the dual-targeting H9 vaccine represents a significant advancement in avian influenza vaccination strategies by enhancing immune responses and providing superior protection. This innovative approach reduces the dependency on higher vaccine doses or additional adjuvants, offering a more efficient and practical solution for disease control. It is particularly suited for preserving antigens in vaccines that are difficult to prepare and of high value, as well as for developing vaccines that can rapidly stimulate immune responses and shorten the immune window. Furthermore, it establishes a robust foundation for the development of next-generation vaccines with broader protection and application across diverse avian pathogens.

## Figures and Tables

**Table 1:** Immunization strategy for the different chicken groups.

Groups	Antigen * (EID_50_ per Chicken)	Immuno-Booster	Adjuvant	Dosage (mL)
H9	1.0 × 10^8^	n/a	ISA 206	0.5
1/2 H9	5.0 × 10^7^	n/a	ISA 206	0.25
1/2 H9+CVCVA5	5.0 × 10^7^	CVCVA5 50 μL per chicken	ISA 206	0.3
1/2 H9+GRFT-VHH54	5.0 × 10^7^	GRFT-VHH54 250 ng per chicken	ISA 206	0.5
1/2 H9+GRFT-VHH74	5.0 × 10^7^	GRFT-VHH74 250 ng per chicken	ISA 206	0.5
Commercial Vac **	≥1.0 × 10^8^	n/a	Mineral oil	0.3
PBS	n/a	n/a	n/a	0.5

* The H9 subtype avian influenza virus was diluted to 2.0 × 10^8^ EID50 per milliliter and inactivated with β-propiolactone (*v*/*v* 0.5‰, 24 h, 37 °C). ** Inactivated H9 type avian influenza vaccine (strain NJ01) was purchased from Zhaofeng Hua Biotechnology (Nanjing, China) Co., LTD Co., and the antigen concentration was ≥ 1.0 × 10^8^ EID50 per 0.3 mL.

**Table 2:** Virus isolation post-challenge.

Groups	Number of Chickens Shedding H9 Virus/Total Number			Number of Sick Chicken Post-Challenge/Total Number *
	3dpc	5dpc	7dpc	
H9	3/10	4/10	1/10	4/10
1/2 H9	5/10	7/10	4/10	7/10
1/2 H9+CVCVA5	1/10	2/10	0/10	2/10
1/2 H9+GRFT-VHH54	2/10	3/10	0/10	3/10
1/2 H9+GRFT-VHH74	1/10	1/10	0/10	1/10
Commercial Vac	2/10	3/10	0/10	3/10
PBS	7/10	10/10	10/10	10/10

* Sick chicken stands for chickens that showed one or more of the following symptoms: depression, anorexia, comb cyanosis, disequilibrium, and death.

## Data Availability

The data can be share up on request.
